# Robustness of Internet of Battlefield Things (IoBT): A Directed Network Perspective

**DOI:** 10.3390/e22101166

**Published:** 2020-10-16

**Authors:** Yuan Feng, Menglin Li, Chengyi Zeng, Hongfu Liu

**Affiliations:** College of Intelligence Science and Technology, National University of Defense Technology, Changsha 410073, China; fengyuan18@nudt.edu.cn (Y.F.); limenglin18@nudt.edu.cn (M.L.); zengchengyi08@nudt.edu.cn (C.Z.)

**Keywords:** internet of battlefield things, complex networks, directed networks, optimal attack strategy, network robustness

## Abstract

Through the combination of various intelligent devices and the Internet to form a large-scale network, the Internet of Things (IoT) realizes real-time information exchange and communication between devices. IoT technology is expected to play an essential role in improving the combat effectiveness and situation awareness ability of armies. The interconnection between combat equipment and other battlefield resources is referred to as the Internet of Battlefield Things (IoBT). Battlefield real-time data sharing and the cooperative decision-making among commanders are highly dependent on the connectivity between different combat units in the network. However, due to the wireless characteristics of communication, a large number of communication links are directly exposed in the complex battlefield environment, and various cyber or physical attacks threaten network connectivity. Therefore, the ability to maintain network connectivity under adversary attacks is a critical property for the IoBT. In this work, we propose a directed network model and connectivity measurement of the IoBT network. Then, we develop an optimal attack strategy optimization model to simulate the optimal attack behavior of the enemy. By comparing with the disintegration effect of some benchmark strategies, we verify the optimality of the model solution and find that the robustness of the IoBT network decreases rapidly with an increase of the unidirectional communication links in the network. The results show that the adversary will change the attack mode according to the parameter settings of attack resources and network communication link density. In order to enhance the network robustness, we need to adjust the defense strategy in time to deal with this change. Finally, we validated the model and theoretical analysis proposed in this paper through experiments on a real military network.

## 1. Introduction

The Internet of Things (IoT) provides a new way for massive deployment of smart devices, such as heterogeneous machines, sensors, and actuators [1,2]. Through ubiquitous connections, devices can exchange data and leverage each other’s information, which enables the Internet of Things to have a high degree of situational awareness [3]. Since the combat efficiency and coordinated decision-making of a modern army depend highly on the ability to have real-time situation awareness on the battlefield and the smooth dissemination of combat information [4,5], the Internet of Things has a promising application in the context of modern warfare. IoT technology used for the interconnection between combat equipment and other battlefield resources is referred to as the Internet of Battlefield Things (IoBT) [6,7]. The collection of real-time data and information dissemination rely on the connectivity of the network, which is vital in allowing the IoBT network to unleash its full potential [8]. However, most of the connections within the IoBT are wireless in nature [9,10,11]. Attackers can selectively destroy specific channels and data packets, causing communication links in IoBT networks to be removed [1,12]. Therefore, considering the presence of malicious attacks by the enemy, the robustness of the IoBT network is crucial to maintaining the combat capability and situational awareness of troops on the battlefield.

Chen et al. studied a two-layer secure network formation problem for IoT networks in which the network designer aims to design a network that can resist different number of link failures with the least resources [1]. Leveraging the theories of stochastic geometry and mathematical epidemiology, Farooq et al. presented a generic framework for secure and reconfigurable design of IoBT networks, which can reconfigure the network according to the changing mission requirements [8]. Farooq et al. also proposed a cognitive connectivity framework, which can cognitively adapt to the changes in the network to interconnect spatially dispersed smart devices thus making it possible to remotely deploy mobile Internet of Things [3]. In order to model and analyze malware infection in wireless IoT networks, Farooq and Zhu proposed a variation of the mean field population process model based on a customized state space that allows us to analyze the formation of botnets in wireless IoT networks and helps in making corresponding network defense decisions [9]. Most of the aforementioned studies are carried out from the perspective of network security design, and there is no research to analyze the robustness of the Internet of Battlefield Things under the optimal attack strategy from the opponent (namely, the ability of the network to maintain connectivity in the worst case).

The network disintegration model in complex network theory has been successfully applied to the modeling and analysis of traditional infrastructure network robustness problems [13,14,15,16,17,18]. By randomly or intentionally removing nodes or edges from the network to simulate random failures or malicious attacks, these models accurately capture the key nodes or edges in the network [19,20,21,22,23]. These research findings will not only be helpful in guiding different protection strategies, but will also be useful in the design of high-robustness and low-cost infrastructure networks [13]. Although the research on the robustness of infrastructure networks has achieved rich results, previous studies did not take the direction of communications between nodes in the network and the optimal attack strategy of the adversary into account, and hence cannot give meaningful insights for the IoBT network. The directionality of communications is an important feature that is not ignorable for IoBT networks. For example, the command and control node will receive data from the sensing node, generate the operation command, and send it to the fire strike node [5,24,25,26]. In this process, the information can only propagate between nodes along the sending direction or receiving direction. For systems with directional connections, the directed network is a network model that naturally describes this communication mode, and the edges in the network have directionality [27,28,29]. Another vital feature of IoBT networks is to make the network connectivity resistant to edge removal attacks by anticipating the worst attack behaviors [1]. From the perspective of attackers, some scholars convert the network disintegration problem into a combinatorial optimization-based problem by introducing some meta-heuristic algorithms to identify the optimal attack strategy [30,31,32,33], which provides an effective method to predict the worst attack behavior of attackers.

In this paper, aiming at the security requirements of the Internet of Battlefield Things, we first use the directed network to model the wireless communication characteristics of the IoBT network. Then, based on the concept of the largest strongly connected component (LSCC) in the directed network, the connectivity of the IoBT network is quantitatively described. Furthermore, we establish an optimization model to simulate the optimal attack strategy of the enemy and verify the optimality of the model solution by comparing it with the disintegration effect of some benchmark strategies. The experimental results show that the network robustness decreases with an increase of the proportion of unidirectional edges in the IoBT network, which means that the number of nodes that can maintain mutual access will decrease rapidly when the IoBT network is attacked maliciously. Furthermore, we also found that under different attack resources of opponents and with changes to the communication link density of the IoBT network, the optimal attack mode of the adversary will change significantly, which enlightens us to adjust the edge protection strategy to deal with the change of adversary attack mode.

The remainder of this paper is organized as follows. In Section 2, we propose a network model and connectivity measurement of the Internet of Battlefield Things. In Section 3, we construct an optimal attack strategy optimization model to simulate the attack behaviour of the enemy. In Section 4, the robustness of the IoBT network under the optimal attack strategy is investigated through experiments on the synthetic networks and real military networks, and give the network defense strategies under different parameter settings. Finally, the conclusions are given in Section 5.

## 2. The Construction of Network Model

**Figure 1:**
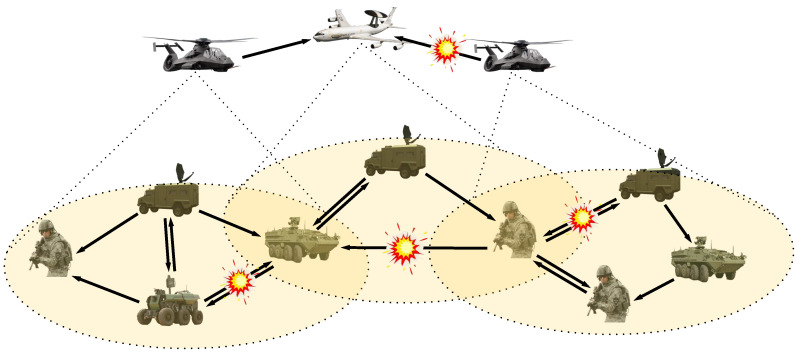
A typical IoBT network with high situational awareness capability. The intelligent devices on the battlefield can exchange data and share critical information through D2D communications, and the directed edges indicate the propagation direction of data or information in the network. The enemy can selectively destroy specific channels and packets, resulting in the deletion of communication links in the network.

In this section, we first use the directed network model to describe the wireless communication characteristics of the IoBT network. Then, based on the concept of the largest strongly connected component (LSCC) in the directed network, the connectivity between devices in the IoBT network is quantitatively described. Figure 1 illustrates a typical battlefield environment comprised of airborne warning aircraft, armed helicopters, armored vehicles, and soldiers, whose communications are threatened by physical or cyber attacks. The enemy can destroy specific channels and data packets, resulting in some equipment being unable to send real-time data or unable to receive the latest operational command, which dramatically reduces the situation awareness ability and combat efficiency of the Internet of Battlefield Things.

### 2.1. Directed Network Model

Systems with asymmetric or unidirectional connections can be described as a directed network, which is represented by a simple directed graph $G=\left(V,E\right)$, and there are no loops and multiple edges in the network. $V=\left\{v_{1},v_{2},\ldots ,v_{N}\right\}$ is the set of nodes and $E=\left\{e_{1},e_{2},\ldots ,e_{W}\right\}$ is the set of directed edges. Let N=|V| be the number of nodes and W=|E| be the number of edges, respectively. Any edge in the network corresponds to a pair of nodes $e_{x}=\left(v_{i},v_{j}\right)$, where node $v_{i}$ is the starting point and node $v_{j}$ is the end point. Denote by $A\left(G\right)={\left(a_{ij}\right)}_{N\times N}$ the adjacency matrix of *G*, in which $a_{ij}=1$ if there is a directed edge from $v_{i}$ to $v_{j}$, and $a_{ij}=0$ otherwise. It should be noted that, unlike undirected networks, the adjacency matrix of directed networks is generally asymmetric. Let $k_{i}^{out}=\sum_{j=1}^{N}A_{ij}$ and $k_{i}^{in}=\sum_{j=1}^{N}A_{ji}$ be the out-degree and in-degree of node $v_{i}$, respectively.

**Figure 2:**
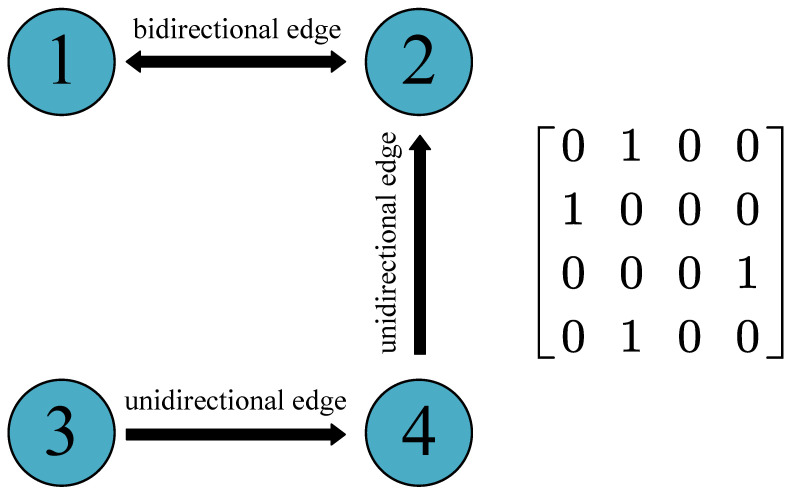
Three edge types between two nodes in a directed network. Node 1 and Node 2 both have edges pointing to each other, so the corresponding edge type is a bidirectional edge. There is only one edge between Node 3 and Node 4, and the corresponding edge type is a unidirectional edge. There is no edge between Node 1 and Node 3. The right side of this figure shows the adjacency matrix, which is an asymmetric matrix.

In this study, we only consider attacks against edges. We define the edge type between any two nodes in the directed network, including no edge, bidirectional edge, and unidirectional edge. The specific definitions are shown in Figure 2. Obviously, three edge types correspond to three possible communication modes between two nodes. If there is no direct communication between the two nodes, such as when affected by geographical factors, it can be represented as no edge. If two nodes send information to each other, it can be represented by a bidirectional edge. A unidirectional edge means that one node can only send or receive information.

### 2.2. Network Connectivity Evaluation Index

**Figure 3:**
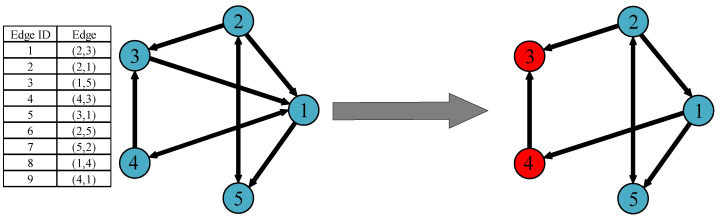
Illustration of the change of the largest strongly connected component LSCC after the directed network is attacked. There is a path between any pair of nodes in the largest strongly connected component. After the two edges (3,1) and (4,1) in the initial network are removed, Node 3 and Node 4 are separated from the largest strongly connected component and become failed nodes (marked in red), while Node 1, Node 2, and Node 5 constitute the new largest strongly connected component (marked in blue).

In this article, to visually observe the impact of edge attack behaviors on IoBT network connectivity, we use the proportion of nodes in the largest strongly connected component (LSCC) as the measure function of network connectivity. For the IoBT network, there is at least one reachable communication path between any pair of combat units in the LSCC. We assume that the larger the LSCC of the IoBT network is, the better the robustness of the network. In Figure 3, we use a simple example to illustrate the change of LSCC after the network is attacked.

## 3. Optimization Model for Edge Attack Strategy in Directed Networks

In this section, we model the optimal attack launched by the enemy on our IoBT network in the battlefield environment. We first define the edges attacked in directed networks. Then, we construct the optimal edge attack strategy optimization model in directed networks and obtain the optimal attack strategy of the enemy by solving the model.

Each edge attack removes an existing edge in the network. If one edge from node $v_{i}$ to node $v_{j}$ is removed, then $a_{ij}=0$, which means that the edge type may be converted from a bidirectional edge to a unidirectional edge, or from a unidirectional edge to no edge. Denote by $\hat{E}\subseteq E$ the set of directed edges that are removed and denote by $\hat{G}=\left(\hat{V},E-\hat{E}\right)$ the network after the edge removal. Denote by $m=\left|\hat{E}\right|$ the attack strength parameter. If m=W, all edges of network *G* are removed. We define an edge attack strategy as $\hat{X}=\left[x_{1},x_{2},\ldots ,x_{W}\right]$, where $x_{i}=0$ if $x_{i}\in \hat{E}$, otherwise $x_{i}=1$. Therefore, a constraint of the edge attack strategy can be obtained as follows:

(1) $$\begin{array}{c} m=W-\sum_{i=1}^{W}x_{i}\;. \end{array}$$

Our goal is to find the optimal edge attack strategy to minimize network performance. The measure function of network performance is denoted by $\Phi \left(\hat{G}\right)$. The problem of solving the optimal edge attack strategy in a directed network could be formulated as follows:

(2) $$\begin{array}{c} s.t.\left\{\begin{array}{c} \sum_{i=1}^{W}x_{i}=W-m,0\leq m\leq W\\ x_{i}=0\;\;or\;\;1,i=1,2,\ldots ,W \end{array}\right. \end{array}$$

For a directed network with *W* edges, the size of the solution space of this problem is $\left(\begin{array}{c} W\\ m \end{array}\right)$, which increases sharply with the size of the network. It is almost impossible to traverse all possible combinations of edges if the directed network’s size is large. Fortunately, the tabu search algorithm has been successfully applied to solve the optimal node attack strategy in undirected networks [30]. The tabu search algorithm, which is one of the meta-heuristic algorithms, is a powerful tool for solving combinatorial optimization problems. It employs a move mechanism and a tabu list to prevent subsequent iterations from falling into a sub-optimal solutions and proposes an aspiration criterion to release some of the better solutions that are currently in the tabu list, thereby ensuring the possibility of an efficient global search. At the same time, we have also noticed that, as a kind of meta-heuristic algorithm, the genetic algorithm is also applied to solve the optimal edge attack strategy in an undirected network [33]. The genetic algorithm has strong global search capabilities, but a simple genetic algorithm not only easily converges prematurely, but also makes it difficult to obtain the global optimal solution [34].

In this work, we compare the disintegration effects of the optimal solutions found by the Tabu Search Algorithm (TS) and Genetic Algorithm (GA). Please refer to these works of literature [30,31,32,33] for the detailed steps and algorithm diagram of the TS algorithm and GA algorithm.

## 4. Experimental Analysis

In this section, we analyze the optimal edge attack strategy based on the optimization model introduced above. On the one hand, we focus on the disintegration effect of the optimal edge attack strategy on the synthetic directed networks. In order to verify that the attack strategy solved by the model can simulate the optimal edge attack behavior, namely, the solution is an approximate optimal solution, we introduce some existing edge attack strategies as benchmarks for comparison. On the other hand, considering the constraint of communication cost on the proportion of bidirectional edges, by setting a certain proportion of bidirectional edges as unidirectional edges at random, we simulate the IoBT network with different communication link densities and study the impact of the change in the proportion of unidirectional edges on the network robustness.

### 4.1. Synthetic Directed Networks

Due to the ubiquity of random connections and power-law characteristics in the real world, in this paper we attempt to employ the directed scale-free network and directed random network as the fundamental network model of the IoBT network. We first generate an undirected scale-free network with a power-law degree distribution $p\left(k\right)=\left(\lambda -1\right)m^{\lambda -1}k^{-\lambda }$. We then randomly select *q* proportion bidirectional edges from this initial network and set them as unidirectional edges to obtain a directed scale-free network. In particular, by defining $E_{q}$ as the total number of directed edges in the directed network under the parameter *q*, it can be derived that $E_{q}=\left(1-q/2\right)\cdot E$. If q=0, then $E_{q}=E$, and the edges in the network are all bidirectional. Additionally, if q=1, then $E_{q}=E/2$, and the edges in the network are all unidirectional. Therefore, the directed scale-free network model is denoted by $SF\left(E_{q},N,m,\lambda ,q\right)$. Similarly, the directed random network can be generated by determining the number of nodes *N* and the probability *p* of connecting edges between any two nodes, which can be indicated by $ER\left(E_{q},N,p,q\right)$.

### 4.2. The Existing Edge Attack Strategies

In order to verify the disintegration effect of the optimal attack strategy obtained by solving the optimization model, as a comparison, we introduce two common methods, namely, Edge-Degree (based on local information) and Edge Betweenness Centrality (based on global information), as benchmarks.

#### 4.2.1. Edge Attack Strategy Based on Edge-Degree

In undirected networks, determining the importance of edges based on the information of two endpoints has been proven to be an effective method in disrupting networks [35]. The degree is a classic local index to measure the importance of nodes. The importance of edges can be defined by the sum or product of the degrees of two endpoints. In this work, according to the concepts of out-degree and in-degree, we define three attack strategies: removing edges in decreasing order of the sum of out-degree (SOD), the sum of in-degree (SID), and the sum of out-degree and in-degree (SOID). These definitions are given by Equation (3).

(3) $$\begin{array}{c} SOD_{e}=k_{i}^{out}+k_{j}^{out}\\ SID_{e}=k_{i}^{in}+k_{j}^{in}\\ SOID=SOD_{e}+SID_{e}\;, \end{array}$$

where $k_{i}^{out}$ and $k_{j}^{in}$ are the out-degree of endpoint *i* and the in-degree of endpoint *j*, respectively. We collectively refer to the definitions in Equation (3) as Edge-Degree. Following the definition of Edge-Degree, the edges that connect the in-hubs or the out-hubs will be attacked preferentially.

#### 4.2.2. Edge Attack Strategy Based on Edge Betweenness Centrality

The Edge Betweenness Centrality quantifies the role of edges in maintaining interconnections between components in the network by reflecting the ratio of the number of shortest paths between nodes *i* and *j* to the total number of shortest paths between all pairs of nodes. Extending this concept directly to directed networks, we define the attack strategy based on Edge Betweenness Centrality (EBC), which removes edges in descending order of betweenness centrality. The definition is given by Equation (4).

(4) $$\begin{array}{c} EBC_{e}=\sum_{i\neq j}\frac{\sigma_{ij}\left(E\right)}{\sigma_{ij}}\;, \end{array}$$

where $\sigma_{ij}$ is the number of shortest paths from node *i* to node *j*, and $\sigma_{ij}\left(E\right)$ is the number of shortest paths from *i* to *j* that pass through edge *E*.

### 4.3. Experiments in Synthetic Networks

We first conducted experiments on both a directed scale-free network and a directed random network with parameters q=0.2,0.5,0.8, corresponding to the three cases where the proportion of unidirectional edges in the directed network is small, half, and large, respectively.

This paper uses the relative size of the largest strongly connected component (LSCC) mentioned in Section 2 as the performance measurement function of the directed network. Denote by f=m/W the directed edge deleting fraction. As the value of *f* increases, the network will eventually collapse, until the relative size of the LSCC almost approaches 0.

The parameters of the optimization model for solving the optimal edge attack strategy based on tabu search were set as the following: the maximum of iterations $T_{\max}=100$, the number of candidates $n_{candidate}=100$, and the tabu length $L_{tabulist}=100$.

The parameters of the optimization model for solving the optimal edge attack strategy based on the genetic algorithm are set as the number of iterations is also set to T = 100; the selection probability is 0.5, the crossover probability is 0.5, the mutation probability is 0.5 and the initial population size is 300.

In order to eliminate the influence of randomness on the experiment as much as possible, the average value of 100 independent experiments was taken as the experimental result.

**Figure 4:**
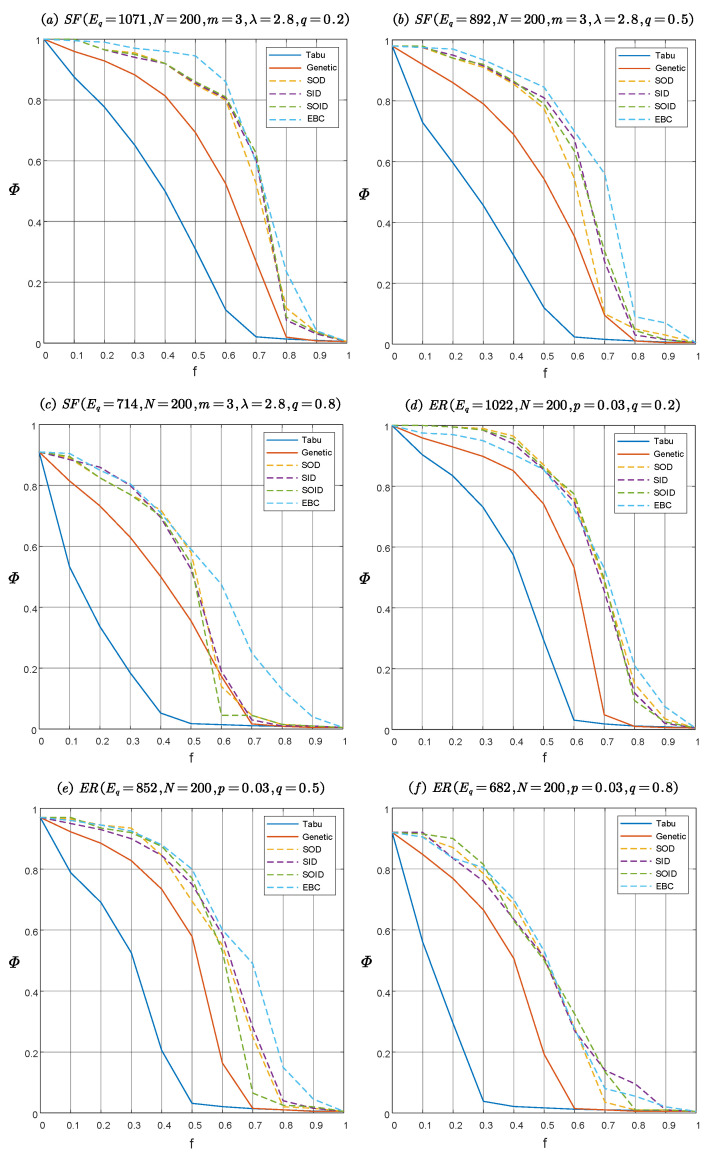
The disintegration effect Φ versus the directed edge deleting fraction *f* with various edge attack strategies in directed scale-free networks $SF\left(E_{q},N,m,\lambda ,q\right)$ and directed random network $ER\left(E_{q},N,p,q\right)$. Tabu, Genetic, SOD, SID, SOID and EBC represent the strategies based on tabu search, genetic algorithm, sum of out-degree, sum of in-degree, sum of out-degree and in-degree, and edge betweenness centrality respectively. To eliminate the influence of randomness on the results, each quantity is an average over 100 independent experiments. (**a**) The disintegration effect on SF network with q=0.2. (**b**) The disintegration effect on SF network with q=0.5. (**c**) The disintegration effect on SF network with q=0.8. (**d**) The disintegration effect on ER network with q=0.2. (**e**) The disintegration effect on ER network with q=0.5. (**f**) The disintegration effect on ER network with q=0.8.

The disintegration effects $\Phi \left(G\right)$ as a function of the directed edge deleting fraction *f* with various edge attack strategies in directed scale-free networks and directed random networks are shown in Figure 4. We found that, given the directed edge deleting fraction, the disintegration effect of the optimal attack strategy solved by the optimization model in Section 3 is much better than other heuristic attack strategies. The results show that the model can simulate the optimal attack launched by the enemy on the IoBT network. In Figure 4, it can be observed that the disintegration effect of the solution obtained by the tabu search algorithm is better than the genetic algorithm, which shows that the solution obtained by tabu search algorithm is closer to the optimal solution than genetic algorithm. Therefore, in the following, only the tabu search algorithm is used to solve the optimization model. In addition, to investigate the influence of communication link density on the robustness of the IoBT network, we set up directed scale-free networks and directed random networks with q=0.2,0.5,0.8, corresponding to high, medium, and low communication link densities, respectively. As can be seen from Figure 4, with an increase of the proportion of unidirectional edges (namely, a decrease of communication link density), the decline curve of network connectivity becomes steeper, which means that the number of nodes in the IoBT network that can maintain mutual accessibility is rapidly decreasing. This shows that the network robustness can be improved by increasing the communication link density.

Table 1 and Table 2 respectively show the standard deviation of the results solved by the two meta-heuristic algorithms in Figure 4. In order to simplify the representation, we only reserved the parameter *q* in the network name as the logo in the bottom right corner.

After successfully predicting the optimal attack strategy, we need to formulate the corresponding defense strategy for our IoBT network to deal with the potential threat from the enemy. To keep the IoBT network resistant to attacks, the network defender can invest in securing its links against failures, where we refer to these specially protected communication edges as protected links [1]. A protected link can be designed by using the moving target defense (MTD) strategy, where the network defender randomly uses communication links among multiple created channels between two nodes [36]. However, considering the cost of these protected links, we can only protect part of the communication edge in the network. Thus, we need to know which possible attack modes are adopted in the optimal attack strategy of the enemy. It is easy to conclude that there are three attack modes that the enemy may take, which are defined as follows: (1) Mode 1: the unidirectional edge is attacked, and the communication between the two nodes is completely cut off; (2) Mode 2: only one unidirectional edge in the bidirectional edge is attacked, and another unidirectional edge is left to communicate between the two nodes; (3) Mode 3: both unidirectional edges in the bidirectional edge are attacked, and the communication between the two nodes is completely cut off. Note that the first two attack modes only need to attack once and remove one edge, while the third attack mode needs to attack twice and remove two edges.

**Figure 5:**
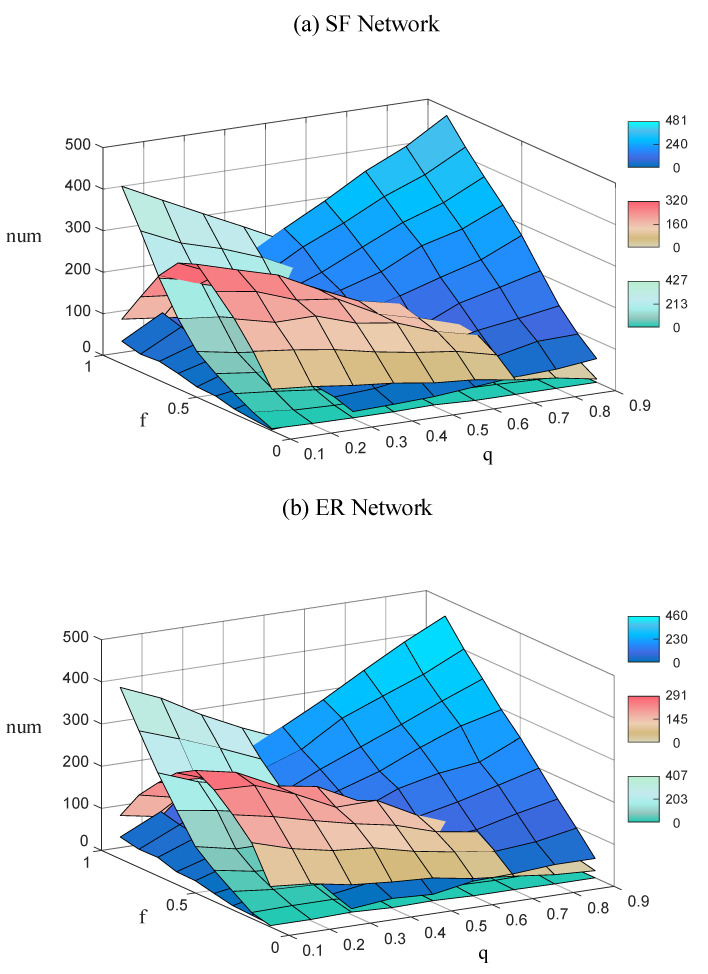
Under the optimal edge attack strategy, the amounts of three typical attack modes will change with the directed edge deleting fraction *f* and the proportion of unidirectional edges *q*, which are Mode 1 (blue color bar), Mode 2 (red-brown color bar), and Mode 3 (cyan color bar). (**a**) Directed scale-free networks $SF\left(E_{q},N=200,m=3,\lambda =2.8,q\right)$ and (**b**) directed random network $ER\left(E_{q},N=200,p=0.03,q\right)$.

Under the parameter settings of f=0.1,0.2,…,0.9 and q=0.1,0.2,…,0.9, we counted the amounts of the three attack modes adopted by the optimal attack strategy. Figure 5 shows the change of amounts of three attack modes adopted by the optimal attack strategy in different situations.

As can be seen from Figure 5, when the enemy attack resources are limited (*f* value is low), and the communication link density of our IoBT network is high (*q* value is low), the enemy tends to use Mode 2 as the primary attack mode. There is a large amount of bidirectional communication in our network, so Mode 3 is not a cost-effective attack mode for an enemy with limited resources. However, the enemy can destroy one unidirectional edge in the bidirectional edge to reduce the proportion of bidirectional edges, which will reduce the possibility of large-scale strongly connected components in the network and thus weaken the combat effectiveness of our IoBT network. Consequently, in this case, we should focus our defense resources on the protection of bidirectional edges. When the enemy attack resources are abundant (*f* value is high) and the communication link density of our IoBT network is high (*q* value is low), the enemy tends to use Mode 3 as the primary attack mode. It is the enemy’s first concern to destroy the network connectivity as much as possible, rather than whether the attack mode is cost-effective. By cutting off the bidirectional communication between massive nodes, the enemy can cut our IoBT network into smaller connected pieces. Similarly, in this case, we should focus our defense resources on the protection of bidirectional edges. No matter whether the attack resources are limited or abundant when our IoBT network communication link density is low (*q* value is high), the enemy will choose Mode 1 as the primary attack mode. Due to the poor robustness of the network with a high proportion of unidirectional edges, the network connectivity will collapse quickly if the enemy attacks the unidirectional edge. Differently from the previous two cases, we should focus our defense resources on the protection of unidirectional edges if our network robustness is poor.

### 4.4. Experiments in Real Military Network

In this section, we use a real military organization network to verify the model and theoretical analysis proposed in this paper. The military network data come from the literature [37], which contains 89 entities, including 12 command nodes, 26 force nodes and 51 intelligence nodes, and there are 150 observable edges. The basic topological features of the military network are shown in Table 3. By setting a certain proportion of bidirectional edges as unidirectional edges at random, we study the network robustness of this military network under different communication link densities. Consistent with Figure 4, we set the unidirectional edge ratio parameters q = 0.2, 0.5, 0.8, corresponding to high, medium and low communication link densities, respectively.

**Figure 6:**
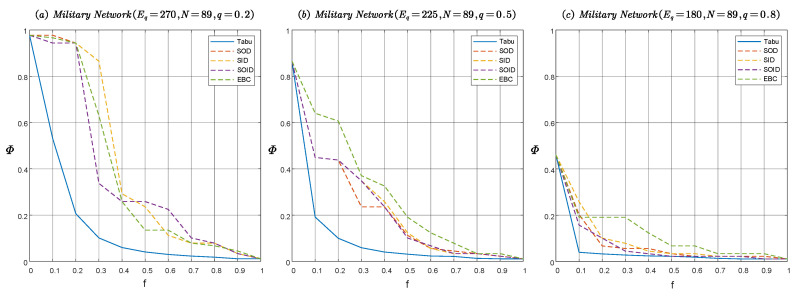
The disintegration effect Φ versus the directed edge deleting fraction *f* with various edge attack strategies in real military networks $Military\;\;Network\left(E_{q},N,q\right)$. Tabu, SOD, SID, SOID and EBC represent the strategies based on tabu search, sum of out-degree, sum of in-degree, sum of out-degree and in-degree, and edge betweenness centrality, respectively. To eliminate the influence of randomness on the results, each quantity is an average over 100 independent experiments. (**a**) The disintegration effect on military network with q=0.2. (**b**) The disintegration effect on military network with q=0.5. (**c**) The disintegration effect on military network with q=0.8.

The disintegration effect Φ versus the directed edge deleting fraction *f* with various edge attack strategies in a real military network are shown in Figure 6. Obviously, the network robustness of this military network is poor. If the enemy takes the optimal attack, then only the removal of 0.2 percent of the edges in the network can make the relative size of the LSCC drop sharply to 0.2 and below. From the perspective of network defense, the topology and number of edges in this military network should be changed to improve the network robustness in the face of possible optimal attacks.

**Figure 7:**
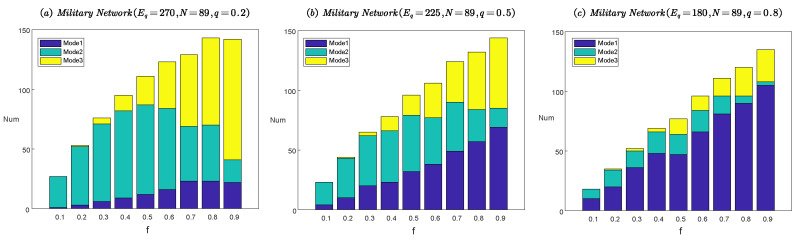
Three attack modes versus the directed edge deleting fraction *f* under the optimal attack strategy in real military networks $Military\;\;Network\left(E_{q},N,q\right)$. Mode 1 represents that the unidirectional edge is attacked. Mode 2 represents that only one unidirectional edge in the bidirectional edge is attacked. And Mode 3 represents that both unidirectional edges in the bidirectional edge are attacked. (**a**) Three attack modes on the military network with q=0.2. (**b**) Three attack modes on the military network with q=0.5. (**c**) Three attack modes on the military network with q=0.8.

Furthermore, we show in Figure 7 the relationship between the number of three attack modes and the directed edge deleting fraction *f* under the optimal attack strategy. To facilitate the reader to visually compare the number changes in the three attack modes, we use a stacked bar plot to display the results.

The results in Figure 7 are consistent with the theoretical analysis in Figure 5. As shown in Figure 7a, when the communication link density is high (q=0.2) and the enemy attack resources are limited (*f* value is low), the enemy will choose Mode 2 as the primary attack mode. As the attack resources increase (*f* value is high), Mode 3 becomes the primary attack mode. As shown in Figure 7b, when the communication link density is medium (q=0.5), with the increase in attack resources, the enemy’s primary attack mode gradually changes from Mode 2 to Mode 1 and Mode 3. As shown in Figure 7c, whether the attack resources are limited or abundant when the communication link density of the military network is low (q=0.8), the enemy will choose Mode 1 as the primary attack mode.

## 5. Conclusions

In this work, we studied the robustness of the Internet of Battlefield Things (IoBT). Since the connectivity between battlefield nodes plays an essential role in delivering real-time data and ensuring situation awareness, the ability to maintain network connectivity under adversary attacks is a critical property for the Internet of Battlefield Things. Aiming at the security requirements of the IoBT network, we first proposed the directed network model and connectivity measurement. Then, we established the optimal attack strategy optimization model and obtained the worst-case attacks by solving the model. Applying the network model, we analyzed the robustness of the IoBT network under the optimal attack strategy, which provided insights into the development of effective protection strategies to ensure the security and reliability of the IoBT network despite adversarial behaviors.

As part of our future work, we will consider the heterogeneity of devices and extend the network model to heterogeneous multi-layer networks. Although the IoBT network is modeled by directed scale-free networks and directed random networks, the structural characteristics and formation mechanism of real IoBT network are still unclear, which makes the network model in this paper unable to reflect all of the characteristics. We will carry out more works on these two directions in the future.

## Figures and Tables

**Table 1:** Standard deviation of results obtained by tabu search algorithm.

	f=0.1	f=0.2	f=0.3	f=0.4	f=0.5	f=0.6	f=0.7	f=0.8	f=0.9
SF${}_{q=0.2}$	0.0134	0.0192	0.0235	0.0272	0.0309	0.03173	0.0053	0.0033	0.0021
SF${}_{q=0.5}$	0.0135	0.0241	0.0256	0.0344	0.0356	0.0076	0.0031	0.0018	0.0024
SF${}_{q=0.8}$	0.0133	0.0217	0.0279	0.0256	0.0062	0.0034	0.0019	0.0020	0.0007
ER${}_{q=0.2}$	0.0123	0.0164	0.0252	0.0300	0.0570	0.0093	0.0040	0.0022	0.0022
ER${}_{q=0.5}$	0.0143	0.0238	0.0340	0.0831	0.0089	0.0045	0.0025	0.0005	0.0019
ER${}_{q=0.8}$	0.0210	0.0572	0.0202	0.0071	0.0040	0.0029	0.0005	0.0022	0.0005

**Table 2:** Standard deviation of results obtained by genetic algorithm.

	f=0.1	f=0.2	f=0.3	f=0.4	f=0.5	f=0.6	f=0.7	f=0.8	f=0.9
SF${}_{q=0.2}$	0.0043	0.0034	0.0088	0.0088	0.0119	0.0142	0.0254	0.0063	0.0025
SF${}_{q=0.5}$	0.0105	0.0142	0.0108	0.0113	0.0153	0.0213	0.0344	0.0012	0.0000
SF${}_{q=0.8}$	0.0117	0.0135	0.0155	0.0161	0.0174	0.0313	0.0053	0.0021	0.0000
ER${}_{q=0.2}$	0.0034	0.0071	0.0078	0.0106	0.0120	0.0263	0.0152	0.0000	0.0022
ER${}_{q=0.5}$	0.0048	0.0119	0.0093	0.0154	0.0256	0.0512	0.0010	0.0000	0.0000
ER${}_{q=0.8}$	0.0078	0.0120	0.0235	0.0324	0.0600	0.0025	0.0000	0.0018	0.0000

**Table 3:** The basic topological features of the military network. Basic topological features include the number of nodes *N*, the number of edges *E*, the average degree $\langle k\rangle$, the average shortest path length $\langle d\rangle$, the assortative coefficient *r* and the clustering coefficient *C*.

Network	*N*	*E*	$\langle k\rangle$	$\langle d\rangle$	r	C
Military Network	89	150	3.483	8.113	0.2754	0.094
